# Exploiting probability density function of deep convolutional autoencoders’ latent space for reliable COVID-19 detection on CT scans

**DOI:** 10.1007/s11227-022-04349-y

**Published:** 2022-02-24

**Authors:** Sima Sarv Ahrabi, Lorenzo Piazzo, Alireza Momenzadeh, Michele Scarpiniti, Enzo Baccarelli

**Affiliations:** Department of Information Engineering, Electronics and Telecommunications, Sapienza University of Rome, Via Eudossiana 18, 00184 Roma, Italy

**Keywords:** Deep convolutional autoEencoder, Kernel density estimation, Reconstruction error, Hidden representation, COVID-19

## Abstract

We present a probabilistic method for classifying chest computed tomography (CT) scans into COVID-19 and non-COVID-19. To this end, we design and train, in an *unsupervised* manner, a deep convolutional autoencoder (DCAE) on a selected training data set, which is composed *only* of COVID-19 CT scans. Once the model is trained, the encoder can generate the compact hidden representation (the hidden feature vectors) of the training data set. Afterwards, we exploit the obtained hidden representation to build up the target probability density function (PDF) of the training data set by means of kernel density estimation (KDE). Subsequently, in the test phase, we feed a test CT into the trained encoder to produce the corresponding hidden feature vector, and then, we utilise the target PDF to compute the corresponding PDF value of the test image. Finally, this obtained value is compared to a threshold to assign the COVID-19 label or non-COVID-19 to the test image. We numerically check our approach’s performance (i.e. test accuracy and training times) by comparing it with those of some state-of-the-art methods.

## Introduction

There is general agreement on chest computed tomography (CT) scans having a potential role in diagnosing COVID-19 and being particularly effective when used as a complement to polymerase chain reaction (PCR) testing [1–5]. Clearly, cheaper testing methods exist, like lateral flow test. Moreover, the radiation dose inflicted to the patient by a scan may be harmful. However, the method can be useful when a different test is unavailable or too expensive or when a quick diagnosis is needed.

Due to its noteworthy detection capabilities, deep learning (DL) is often employed to assist the evaluation of CT scans for the diagnosis of COVID-19. Indeed, *classification* of chest CT scans is an important and active research area. In recent contributions, classification is carried out by exploiting supervised DL, using only a few classes of chest CTs (for instance, COVID-19 class and normal one). However, the symptoms of chest diseases manifest themselves in a *broad* spectrum of visual characteristics. Hence, supervised-trained models could run into trouble when they are fed with chest CTs that do not belong to any of the classes used in the training phase. This issue can be addressed by taking advantage of unsupervised DL, where the neural network models are trained *only* on data sets of the COVID-19 class. In this manner, the trained model can differentiate the COVID-19 class (the target class) from any other types of chest images (anomalies).

This paper develops an unsupervised classification approach based on autoencoders (AEs). An AE is composed of an encoder and a decoder [6]. The encoder takes an image and transforms it into a hidden feature vector, a compressed input representation. The decoder is used to reconstruct the input image from the hidden feature vector. Since deep convolutional neural networks (CNNs) have proved their effectiveness in image processing and feature extraction, in this paper, we design a deep convolutional autoencoder (DCAE) as the neural network model [7].

We train the proposed DCAE, in an unsupervised manner, on a data set of chest CT scans obtained from COVID-19 patients. After training, the encoder is used to obtain the hidden feature vectors of the training set CT scans. These hidden feature vectors are, in turn, exploited to estimate the probability density function (PDF) of COVID-19 hidden feature vectors, by means of the multivariate kernel density estimation (KDE) method. The classification of a test image is performed by feeding it into the trained encoder to produce its hidden feature vector. Afterwards, the PDF is used to compute the PDF value of the test image. Finally, the obtained PDF value is compared to a suitably tuned threshold to classify the test image as either COVID-19 or non-COVID-19.

For comparison, we also consider a second method for classification based on the DCAE reconstruction error. The resulting error is noticeably higher than the average error corresponding to the training set instances when the trained DCAE is fed with a CT scan of a non-COVID-19 case and attempts to recover it. If this error is below a suitably tuned threshold, the image is classified as COVID-19, otherwise as non-COVID-19.

The rest of the paper is organised as follows: In Sect. 2, we review the related work and present the contributions of this paper. In Sect. 3, we provide details of the used data sets and pre-processing tasks. Sections 4 and 5 describe the proposed DCAE architecture and classification approach, respectively. We discuss the obtained numerical results in Sect. 6 and compare the performance of our approach to the ones of some state-of-the-art approaches that rely on the supervised training method. Finally, in Sect. 7, we point out the summarised observations and provide some hints for future research.

## Related work and paper contributions

The literature on the application of DL-based algorithms to the detection of COVID-19 is vast. The small volume of available data on COVID-19 patients has motivated the researchers to take this deficiency into account. For instance, the transfer learning approach is adopted in [8–17] to deal with the lack of large-size data sets. In [18], the authors utilise GoogleNet and ResNet for supervised COVID-19 classification. The authors of [19] take a statistical method to address issues like huge computational complexity and large datasets required by deep networks. The adopted approach is based on the evaluating and comparing the statistical representation of medical images. The authors of [20] consider an unbalanced-data supervised algorithm and obtained good results comparable with benchmark architectures.

A large number of research papers adopt supervised learning approaches [21–31]. In [21], the authors consider a binary classification problem and apply the off-the-shelf VGG-16. In [26], the authors use depth-wise convolutions with varying dilation rates to extract more diversified features of chest images. The authors use a pre-trained model and reach the overall $90.2 \%$ accuracy. In [28], the authors design a neural network model as a combination of convolutional and capsule layers, called COVID-FACT. Despite their great effort, the considered model achieves the $90.82 \%$ accuracy. The authors of [29] propose a model based on the pre-trained ResNet50 and achieve the accuracy, rather similar to the original ResNet50. The DenseNet-based approach, considered in [31], achieves $92\%$ accuracy. The work by [32] compares different classification architectures on a specific dataset to discover the most suitable real-world scenarios.

The approaches developed in all these papers typically deal with only a limited class of chest images. Hence, a naturally posed question is: to which category does a CT fit when it does not belong to any of the classes learned by the supervised-trained models? In any case, the aforementioned supervised methods must be trained on both COVID-19 and non-COVID-19 CTs.

**Table 1:** A synoptic overview of main related work on COVID-19 detection/classification

Work	Algorithm specifications	Accuracy
Ref. [8]	$\bullet \;$ Modified inception	0.8502
	$\bullet \;$ BC (Pneumonia, COVID)	
	$\bullet \;$ Transfer learning	
Ref. [10]	$\bullet \;$ ResNet50V2/Xception + Softmax	0.9849
	$\bullet \;$ BC (Normal, COVID)	
	$\bullet \;$ Transfer learning	
Ref. [13]	$\bullet \;$ AlexNet/GoogleNet/ResNet + Softmax	0.9905
	$\bullet \;$ MC (Normal, COVID, Tumour)	
	$\bullet \;$ Transfer learning	
Ref. [16]	$\bullet \;$ Modified AlexNet	0.9410
	$\bullet \;$ BC (Normal, COVID)	
	$\bullet \;$ Transfer learning	0.9410
Ref. [17]	$\bullet \;$ DensNet-121	0.8711
	$\bullet \;$ MC (Normal, COVID, Cancer)	
	$\bullet \;$ Transfer learning	
Ref. [18]	$\bullet \;$ AlexNet/GoogleNet/VGG-16	0.9951
	$\bullet \;$ MC (COVID, Atypical/Viral Pneumonia)	
	$\bullet \;$ Transfer learning	
Ref. [20]	$\bullet \;$ Deep CNN	0.9943
	$\bullet \;$ MC (Normal, COVID, Bacterial Pneumonia)	
Ref. [21]	$\bullet \;$ SRGAN/VGG-16	0.9800
	$\bullet \;$ BC (Normal, COVID)	
Ref. [25]	$\bullet \;$ Deep CNN + SVM	0.9866
	$\bullet \;$ MC (Normal, COVID, Bacterial Pneumonia)	
Ref. [29]	$\bullet \;$ ResNet50 + FPN	0.9300
	$\bullet \;$ MC (Normal, COVID, Bacterial Pneumonia)	
Ref. [31]	$\bullet \;$ DenseNet	0.9500
	$\bullet \;$ BC (Normal, COVID)	
Ref. [33]	$\bullet \;$ SAE + Softmax Classifier	0.9470
	$\bullet \;$ BC (Normal + COVID)	
Ref. [34]	$\bullet \;$ Denoising AE + Hidden Features	–
	$\bullet \;$ MLC (COVID, Pneumonia, and other classes)	
Ref. [35]	$\bullet \;$ AE + FPN + Classifier	–
	$\bullet \;$ MC (Normal, COVID, Pneumonia)	
Ref. [36]	$\bullet \;$ VAE + AWF	0.9920
Ref. [37]	$\bullet \;$ DDCAE + Latent Space	1.0000
	$\bullet \;$ (Pneumonia & Normal)	

BC, Binary classification; MC, Multiclassification; MLC, Multilabel classification; UC, Unary classification

Lastly, we provide an overview of AE-based approaches. The authors of [33] consider a stacked AE (SAE), composed of four CAEs, followed by a dense layer, and a final softmax classifier. Each layer is equipped with regularisation to improve the local optimum. The binary classification task, considered by the authors, occurs at the last stage, where the softmax classifier obtains the probability of the two types of labels and performs the classification task with an average accuracy of $94.7 \%$. This method is different from our approach because we perform the classification by directly using the PDF of the hidden feature vectors, without inserting any classifier at the top of our model. The deep convolutional denoising AE, proposed in [34], is trained on COVID-19, pneumonia, and a few other types of chest X-rays. Then, the hidden feature vector of a test image is compared to the features of the selected training data sets. The considered AE exhibits good performance. However, unlike our work, this approach relies on training the considered model over each selected class and therefore cannot detect chest CTs except those that belong to the classes of training data sets. The work by [35] focuses on a two-stage learning method and a triple classification task. The authors train their considered model on classes of COVID-19, pneumonia, and normal cases separately. Once the hidden feature vectors of all classes are independently obtained, a feature classifier is employed and trained**—**in a supervised manner**—**to detect each decision class. The considered approach reaches a quite good accuracy of $93.50 \%$. In contrast to this work, we train our DCAE model on only one class, i.e. the COVID-19. The paper by [36] is based on a variational autoencoder (VAE) model for COVID-19 classification. The VAE model involved adaptive Wiener filtering (AWF)-based pre-processing technique to enhance the image quality. Besides, Inception v4 with Adagrad technique is employed as a feature extractor and unsupervised VAE model is applied for the classification process. As the last research paper, the method introduced by [37] builds a robust statistical generating target histogram of the deep denoising convolutional autoencoder’s (DDCAE) latent vector. It then estimates the statistical distance between unknown and target histograms to classify the images according to proper thresholds.

A brief overview of the given literature is provided in Table 1. Finally, the main contributions of this paper are listed below:

- we base the classification task on exploiting an unsupervised deep neural network model, which is trained only on COVID-19 images and, in so doing, we strengthen the robustness of the proposed model concerning the presence of CT scans of other diseases which are not seen during training phase;
- we propose an *ad hoc* DCAE with an optimised number of layers for the best classifying test performance;
- as an additional novelty, we base the classification task on the estimation of the probability density of the training hidden feature vectors by adopting the KDE method; and finally,
- we carry out numerical tests under benchmark data sets available in the literature and compare the performance of our approach to the ones of some supervised/unsupervised deep neural networks, both in terms of test accuracy and processing times.

## Utilised data sets and pre-processing

The training data set used in this paper is composed of 4000 CTs of COVID-19 cases collected from over 500 patients. These CT scans have been selected from the ‘COVIDx CT-2A’ data set; the ‘A’ variant of the ‘2nd’ version of the open-source ‘COVIDx-CT’ data set [38]. The ‘COVIDx CT-2A’ data has been validated by medically qualified personnel. The data are split into training and validation sets containing $80\%$ and $20\%$ of instances, respectively.

**Table 2:** Train/validation/test set compositions and related web links; WP: Web Page

Data set	COVID-19	Pneumonia	Cancers	Normal	Link
Training	3200	${{-}{-}{-}}$	${{-}{-}{-}}$	${{-}{-}{-}}$	${\hbox {WP1}}^{1}$
Validation	800	${{-}{-}{-}}$	${{-}{-}{-}}$	${{-}{-}{-}}$	WP1
Test	2500	300	900	300	WP1 & ${WP2}^{2}$

^1^ https://kaggle.com/hgunraj/covidxct

^2^ https://kaggle.com/mohamedhanyyy/chest-ctscan-images

The test data set comprises 4000 CT slices of COVID-19, normal, pneumonia, and three types of lung cancers, namely adenocarcinoma, large cell carcinoma and squamous cell carcinoma. The test images have been randomly selected from two separate data sets [38, 39], composed of CTs of over 500 patients different from those involved in the training set. The data sets include both male and female patients and cover a wide age range.

**Fig. 1:**
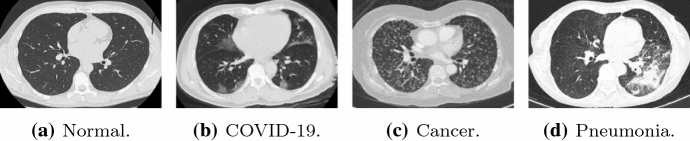
Samples of four lung CT scans drawn from the used training and test data sets

The information about the employed training/test data sets is summarised in Table 2, where we also give the links to the corresponding web pages. For illustrative purpose, in Fig. 1, we present some instances of various chest CT categories, which are drawn from training and test data sets. As can be seen from the figure, the COVID-19 image is characterised by the presence of several opacities. Indeed, commonly reported imaging features specific of COVID-19 pneumonia are peripheral, bilateral, ground-glass opacities with or without visible intralobular lines: see [40] for a wider discussion.

**Fig. 2:**
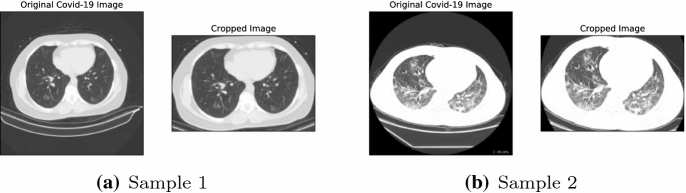
Two samples of original COVID-19 CTs and their cropped versions

As a pre-processing task, the margin of every CT is cropped before performing the training phase. Moreover, all the images are resized to 300 pixels in width and 200 pixels in height: this size was selected as a compromise between computational complexity and resolution. Two samples of the COVID-19 dataset in the original, cropped and resized versions are demonstrated in Fig. 2.

## The proposed DCAE architecture

**Fig. 3:**
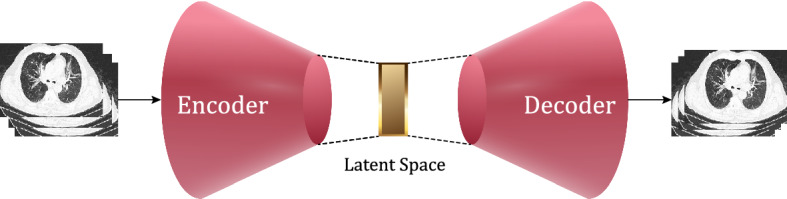
The autoencoder architecture

A DCAE is composed of an encoder and a decoder as depicted in Fig. 3. The encoder takes an image as input and transforms it into a hidden feature vector called latent space. The decoder takes the hidden feature vector and attempts to recover the input image. The difference between the input and the output images results in the reconstruction error, which is the cost function to minimise during the training phase. We point out that the hidden feature vectors have fewer dimensions than those of the input images since the DCAE produces compressed versions.

The DCAE, exploited in this work, has been designed to take account of the trade-off between test classification accuracy and model complexity. Its “*ad hoc*” designed architecture is detailed in Table 3: the encoder is composed of three convolutional layers, two batch normalisation layers, two max-pool layers, followed by flatten and dense layers with rectifier activation functions (ReLUs).

**Table 3:** The architecture of proposed DCAE; encoder input shape: $200 \times 300 \times 3$; decoder input shape: 128; number of parameters of the encoder: 31, 097, 408; number of parameters of the decoder: 31, 337, 475

Layer	Kernel	Stride	Output shape
*Encoder*			
Conv2D	$3\times 3$	–	$200\times 300\times 256$
BatchNorm	–	–	$200\times 300\times 256$
Conv2D	3$\times 3$	–	$200\times 300\times 128$
MaxPool2D	2$\times 2$	–	$100\times 150\times 128$
Conv2D	3$\times 3$	–	$100\times 150\times 64$
BatchNorm	–	–	$100\times 150\times 64$
MaxPool2D	2$\times 2$	–	$50\times 75\times 64$
Flatten	–	–	240000
Dense	–	–	128
*Decoder*			
Dense	–	–	240000
Reshape	–	–	$50\times 75\times 64$
Conv2DTranspose	$3\times 3$	2	$100\times 150\times 128$
BatchNorm	–	–	$100\times 150\times 128$
Conv2DTranspose	3$\times 3$	2	$200\times 300\times 256$
BatchNorm	–	–	$200\times 300\times 256$
Conv2DTranspose	3$\times 3$	1	$200\times 300\times 3$

**Table 4:** Main parameters of the carried out training phase

Description	Value
Batch size	16
Number of epochs	100
Optimiser	Adam
Learning rate	$1 \times 10^{-5}$
Loss function	MSE
Size of hidden feature vectors	128

**Fig. 4:**
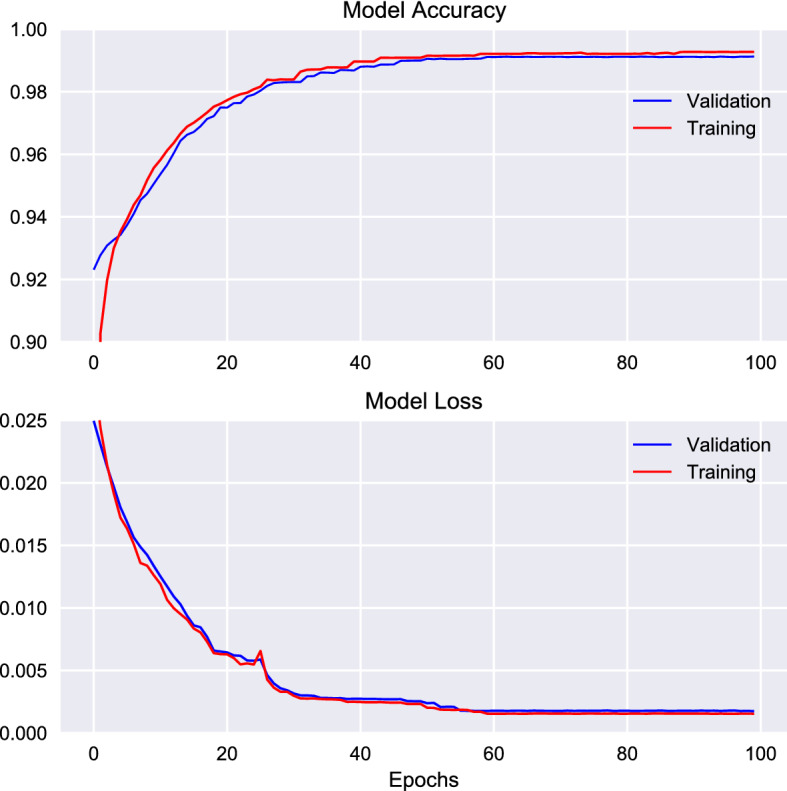
Numerically evaluated plots of accuracy-vs.-epochs and MSE-vs.-epochs under the training phase

The DCAE is trained to recover its input, i.e. COVID-19 CT scans. As training cost function, we use the mean squared error (MSE), and in order to minimise the considered cost function over the training set, we employ the Adam (Adaptive moment estimation) solver, which is a gradient-based stochastic optimisation algorithm that takes account of the first and second moments of underlying gradients [41]. The training is carried out over 100 epochs. After the training phase, we select the DCAE weights, which give rise to the minimum squared error on the validation data set. Table 4 recap details on the adopted model setting. For illustrative purpose, Fig. 4 presents the graphs of MSE and accuracy versus the number of epochs, over which the training phase is carried out.

Note that we have considered other options for both the architecture and the training parameters, using several alternatives proposed in the literature. The various options have been compared by means of unreported tests: the final architecture and training parameters have been selected because they yielded the highest classification accuracy.

The DCAE is implemented in Python language, using TensorFlow and Keras API. All the numerical trials have been carried out on a PC equipped with an AMD Ryzen 9 5900X 12-Core 3.7 GHz processor, two GeForce RTX 3070 graphics cards, and 128 GB RAM.

## Image classification

In this section, we describe the classification approach that is based on the PDF estimation of the DCAE hidden feature vectors.

### PDF estimation of the training hidden feature vectors

In order to estimate the PDF of the hidden feature vectors, a first choice that needs to be done is whether to use a parametric or a nonparametric method. Since we have no clues on the shape of the pdf and we do not want to polarise the estimation by guessing, we decided to use a nonparametric estimate. Among the nonparametric methods, we selected the KDE approach,^1^ which is well known [42] and outperforms the simpler histogram approach.

**Fig. 5:**
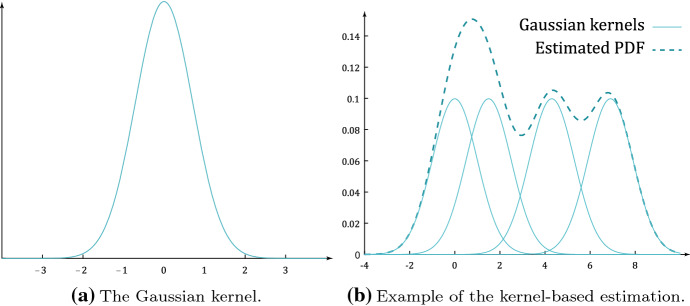
An example of KDE estimation over $n=4$ data points: a copy of the kernel is placed on each data point and the copies are summed to produce the final PDF estimate

The KDE method is based on a univariate kernel function denoted by *K*(*x*). Although a wide variety of different kernels can be used, according to [6], we consider the Gaussian one, i.e. $K(x) = e^{-x^2}$(see Fig. 5a). The kernel is used as an interpolating function to build up the PDF estimate.

In order to describe the KDE approach, we first illustrate it for the simple case of a univariate PDF. Let us consider a set of *n* real numbers: $x_i$ for $i = 1, ..., n$, drawn from a (hidden) random variable (RV) *X*, which exhibits an unknown PDF, $f_X\left( x\right)$, that we want to estimate. The KDE estimate of the PDF is [6]:

1 $$\begin{aligned} {\bar{f}}_X(x)= \frac{1}{\alpha } \sum _{i=1}^{n} K \left( \frac{x-x_i}{h} \right) \end{aligned}$$

where the constant $\alpha$ is a normalisation factor, used to set the integral of ${\bar{f}}_X(x)$ to one, while the parameter *h* is the kernel bandwidth which is used to set the width of the kernel. The estimation is illustrated in Fig. 5b, where we see that the density is obtained as the superposition of *n* scaled and shifted copies of the kernel.

To describe the multivariate case, let us assume that we have a *p* dimensional RV *X*, with a multivariate density $f_X(\mathbf{x})$ where $\mathbf{x} \in {\mathbb {R}}^p$ is a *p*-dimensional vector. Moreover, we have a set of vectors $\mathbf{x}_i \in {\mathbb {R}}^p$ for $i = 1, ..., n$ which are samples drawn from the RV *X*. The KDE estimate generalises to the multivariate case as in:

2 $$\begin{aligned} {\bar{f}}_X(\mathbf{x})= \frac{1}{\alpha } \sum _{i=1}^{n} K \left( \frac{||\mathbf{x}-\mathbf{x}_i||}{h} \right) , \end{aligned}$$

where ||.|| denotes the Euclidean norm.

In order to estimate the PDF of the DCAE hidden feature vector when the input is a COVID-19 image, we use the KDE estimate of (2), where the vectors $\mathbf{x}_i$ are the hidden feature vectors obtained from the images of the training set, with $n=3200$ and $p=128$ in our setting. For the computation of the KDE estimate, we used Scikit-learn [43], an open-source machine learning library developed for the Python environment. One parameter that needs to be carefully tuned is the bandwidth *h*. Indeed, the choice of bandwidth controls the bias–variance trade-off in the estimation of the PDF [6]. This means that a too narrow bandwidth leads to a high-variance estimate (over-fitting), while a too wide bandwidth results in a high-bias estimate (under-fitting). For selecting the bandwidth, we employed the Scikit-learn built-in method of grid search cross-validation. This algorithm selects the best option from a grid of parameter values provided by the user, automating the ‘trial-and-error’ method. A second option could be to use the maximum likelihood cross-validation (MLCV) approach, introduced in [44, 45].

We point out that the computational complexity of the evaluated KDE-based estimation approach depends on the length *p* of the hidden feature vectors and the number *n*, of employed training images. specifically, since *n* Euclidean distances among *p*-dimensional vectors must be computed, the resulting computational complexity scales as:

3 $$\begin{aligned} {\mathcal {O}} \left( n \times p \right) . \end{aligned}$$

### Classification based on the estimated PDF

In our approach, the estimated PDF of the DCAE latent space is used to classify the test images. To this end, we feed the image to the DCAE encoder to produce the corresponding hidden feature vector. Next, the obtained vector is used as the argument of the estimated PDF, in order to compute its corresponding PDF value. In practice, since the obtained values of the PDF are minimal, it is more robust to work with *log probability densities*. If the obtained value of log density is above a (suitably tuned) threshold, the image is classified as a COVID-19 case; otherwise, it is labelled as a non-COVID-19 one.

**Fig. 6:**
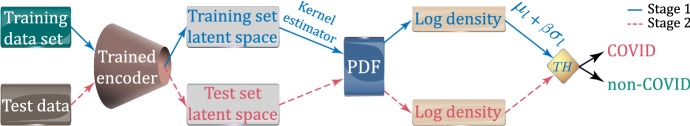
Estimation of the PDF of the latent space (stage 1) and classification of the test images (stage 2)

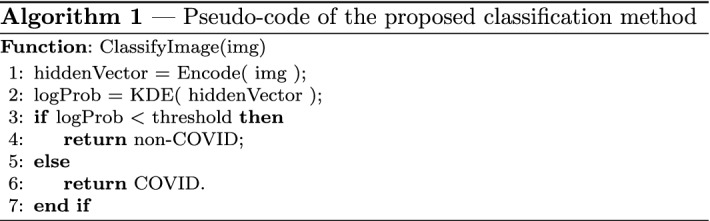



In order to suitably set the decision threshold, we evaluate the real-valued log probability densities of *n* images in the training set, denoted by $l_i$, for $i = 1, 2, \cdots , n$. Then, we compute the mean and the standard deviation of the evaluated $l_i$s, denoted by $\mu _l$ and $\sigma _l$ , respectively. Thus, the threshold is set to:

4 $$\begin{aligned} T\!H = \mu _l + \beta \sigma _l, \end{aligned}$$

where the constant $\beta$ is evaluated by using the validation set. In particular, we select $\beta$ so that the threshold equals the minimum log probability of the validation set, namely $-233.5$: in this way the whole validation set is classified as COVID-19. The overall proposed classification procedure is summarised in Fig. 6 and Algorithm 1.

### A benchmark classifier

For comparison purposes, we consider a benchmark classification method. To this end, we recall that the DCAE is trained to produce an output similar to the input as much as possible. The difference between the input and the output is the reconstruction error, which minimises the cost function during the training phase. However, since the training set contains COVID-19 scans only, the DCAE is effective at recovering COVID-19 images, but it is ineffective for recovering non-COVID-19 images. Therefore, a high reconstruction MSE can be an index that the image does not belong to the COVID-19 class.

**Fig. 7:**
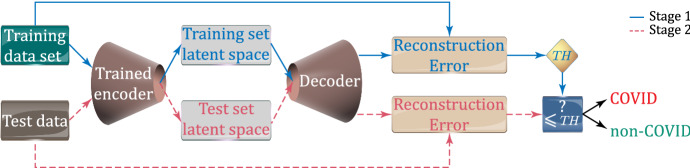
Evaluation of reconstruction errors of training set and the threshold (stage 1), and classification of the test images (stage 2)

By considering this fact, we build up the following benchmark classification procedure. Given an image, we feed it into the DCAE and compute the Euclidean norm of its reconstruction error. If the obtained error norm is below a (suitably) set threshold, the image is classified as COVID-19. The threshold is evaluated by using the instances of validation set. In particular, we select the threshold equal to the maximum error of the validation set, namely 0.08: in this way the whole validation set is classified as COVID-19. Figure 7 presents the flow diagram of the classification process based on reconstruction error.

## Numerical results and performance comparisons

In order to evaluate the performance of our classification method, we carried out several tests. We split the presentation into three parts that are: (i) the results that are obtained from the KDE probabilistic approach; (ii) the results that are achieved through reconstruction error evaluation; and (iii) performance comparisons with some state-of-the-art solutions. In the next subsection, we describe the employed performance metrics as a preliminary step.

### Performance metrics

Given a binary classifier, the considered performance metrics are the rates of true-positive (TP), true-negative (TN), false-positive (FP) and false-negative (FN) assignments. These metrics are summarised in Table 5. These basic metrics can be represented in a compact form as the four elements of the resulting confusion matrix [46].

**Table 5:** Basic metrics

Name	Description
True positive (TP)	COVID-19 image classified as COVID-19
True negative (TN)	Non-COVID-19 image classified as non-COVID-19
False positive (FP)	Non-COVID-19 image classified as COVID-19
False negative (FN)	COVID-19 image classified as non-COVID-19

From these basic metrics, a number of affiliated performance indexes can be derived [46]. This paper will consider accuracy, recall, precision and F1-score, as performance indexes. The formal definitions of these indexes are given in Table 6.

**Table 6:** Utilised performance indexes

Metrics	Formula
Recall	$TP/(TP+FN)$
Precision	$TP/(TP+FP)$
F-score	$2TP/(2TP+FP+FN)$
Accuracy	$(TP+TN)/(TP+FN+FP+TN)$

### Performance of the proposed approach

**Fig. 8:**
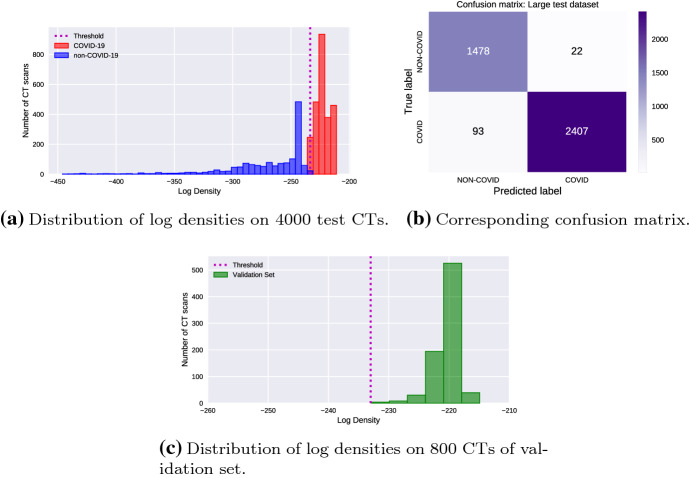
Results under the proposed approach

As a first experiment, we carried out a classification of the test data set by using the proposed approach. To this end, each test image is fed to the trained DCAE, and the corresponding hidden feature vector is obtained. Afterwards, the obtained hidden feature vector is used as an argument of the multivariate PDF estimated by the KDE and then the corresponding log density value is computed. The so-obtained log densities are plotted in Fig. 8a for all the test images and in Fig. 8c for validation set.

From Fig. 8, we see that the log densities of the COVID-19 images are almost separated from those of the non-COVID-19 ones. In Fig. 8a and c, the vertical dashed line denotes the threshold, which is laid between the two classes. As a result, the proposed method achieves a $97.12\%$ test accuracy. The corresponding confusion matrix is presented in Fig. 8b. The last row of Table 7 reports the evaluation performance metrics.

### Performance of the benchmark classifier

**Fig. 9:**
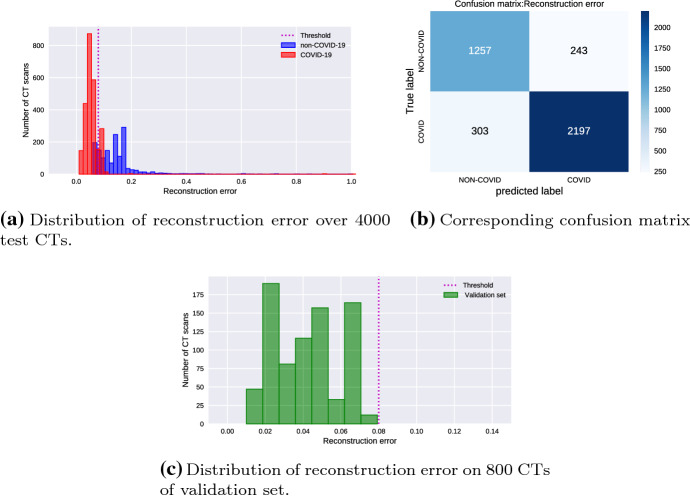
Performance results of the reconstruction error approach

As a second experiment, we evaluate the performance of the benchmark classification approach of Sect. 5.3, which is based on the reconstruction error. To this end, in Fig. 9a, we plot the reconstruction errors obtained by feeding the whole test set to the DCAE, using two different colours for the COVID-19 and non-COVID-19 scans. From the figure, we see that, as expected, the error is lower for the COVID-19 images. However, the two classes are not disjoint. The threshold is shown in Fig. 9a by the vertical dashed line. The resulting accuracy is equal to $86.35\%$. The corresponding confusion matrix is presented in Fig. 9b, and the related performance indexes are reported in Table 7. From Table 7, we conclude that the obtained performance of the classification approach based on the reconstruction error is about $11\%$ inferior performance than the corresponding one of the KDE-based proposed approach.

**Table 7:** Performance metrics of the proposed approach and the benchmark one based on the reconstruction error

Method	Accuracy	Precision	Recall	F1-score	Test CTs
Reconstruction error	0.8635	0.8584	0.8531	0.8555	4000
Our approach	0.9712	0.9741	0.9659	0.9696	4000

### Performance comparison against state-of-the-art approaches and robustness test

In this subsection, we study the classification accuracy of several other approaches and compare their results with ours. For computational complexity reasons, we do not use the whole test set but a subset of it, comprising 1000 images (500 COVID, 120 pneumonia, 120 normal and 260 cancer). On this reduced set, our approach reaches a $100 \%$ accuracy, while the reconstruction error approach reaches $97 \%$ accuracy (see Table 8).

In order to compare our results with those obtained by supervised learning, we have considered three state-of-the-art supervised models, namely GoogLeNet [47], AlexNet [48], and ResNet18 [49], which are typically used for image classification and able to successfully classify out-of-sample examples.

As a first experiment, we have trained—in a supervised way—the aforementioned models, using a training set composed of 2000 COVID-19 CTs and 2000 non-COVID-19 CTs. The non-COVID-19 set is composed of five classes, namely normal CTs, pneumonia and three types of lung cancers CTs. Afterwards, each model has been evaluated on the reduced test set. The obtained performance indexes are reported in Table 8. These results are comparable to those obtained from the KDE-based approach.

**Fig. 10:**
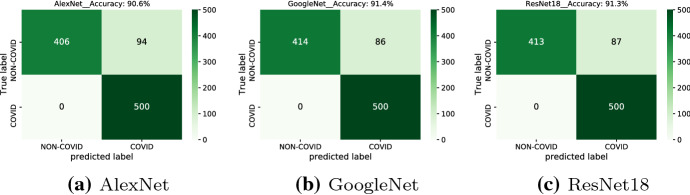
Confusion matrices: pneumonia CTs are present in test data set, but not in the train data set

While the supervised DL models have a performance similar to our approach, we expect that their performance is more sensitive (i.e. less robust) to unseen test images. To corroborate this statement, we carry out a final experiment, where we retrain all the supervised models using a modified data set: we eliminate pneumonia CTs from the non-COVID-19 images, replace them with the normal CTs, apply all the procedures from scratch. Once the best weights are achieved, we perform the test phase on the reduced test set, including pneumonia CTs. The corresponding performance indexes are presented in Table 9, while the confusion matrices are shown in Fig. 10. It is observed that the supervised models are able to distinguish COVID-19 perfectly, but since the pneumonia images have not been included in the training phase, the models are in trouble with these images. In other words, if some classes of images are not present in the training set, the supervised-trained models are not capable of correctly classifying them in the test phase. We conclude that our approach is more robust in the presence of outliers in the test set.

**Table 8:** Comparison with supervised classification

Model	Accuracy	Precision	Recall	F1-score
AlexNet	0.9840	0.9840	0.9841	0.9840
GoogleNet	0.9960	0.9960	0.9960	0.9960
ResNet18	0.9930	0.9931	0.9930	0.9930
Unsupervised approach				
Reconstruction error	0.9710	0.9710	0.9710	0.9710
Our approach	1.0000	1.0000	1.0000	1.0000

The training set is composed of 4000 images (COVID only for the proposed method and belonging to five different classes for the supervised methods). The test set is composed of 1000 images (500 COVID, 120 pneumonia, 120 normal and 260 cancer)

**Table 9:** Robustness tests: pneumonia CTs are present in the test set, but not in the training set for the AlexNet, GoogleNet and ResNet18

Model	Accuracy	Precision	Recall	F1-score
AlexNet	0.9060	0.9060	0.9209	0.9052
GoogleNet	0.9140	0.9140	0.9266	0.9134
ResNet18	0.9130	0.9130	0.9259	0.9123
Unsupervised approach				
Reconstructio error	0.9710	0.9710	0.9710	0.9710
Our approach	1.0000	1.0000	1.0000	1.0000

The test set is composed of 1000 images (500 COVID, 120 pneumonia, 120 normal and 260 cancer)

As a second experiment, we compare our approach with several other methods presented in [37]. In particular, we consider the Histogram-Based DDCAE (HB-DDCAE) method proposed in that work, together with several shallow methods. The shallow methods are: support vector machine (SVM) with 2-degree (SVM-2D) and 3-degree (SVM-3D) polynomial, and radial basis function (SVM-RBF) kernels; multilayer perceptron (MLP) equipped with single hidden layers composed by 50 (MLP-50), 100 (MLP100), and 200 (MLP-200) neurons; random forest (RF) composed by 100 (RF-100), 500 (RF-500), and 1000 (RF-1000) binary trees. See [37] for details about the training. The results are presented in Table 10.

**Table 10:** Comparative analysis of binary classification of COVID-19 vs non-COVID-19 based on similar datasets

Model	Accuracy	Precision	Recall	F1-score
HB-DDCAE	1.0000	1.0000	1.0000	1.0000
SVM-2D	0.9340	0.9417	0.9340	0.9378
SVM-3D	0.9270	0.9363	0.9270	0.9316
SVM-RBF	0.9170	0.9788	0.9170	0.9469
MLP-50	0.7600	0.8132	0.7600	0.7857
MLP-100	0.7740	0.7874	0.7740	0.7806
MLP-200	0.7830	0.8123	0.7830	0.7974
RF-100	0.7110	0.7852	0.7110	0.7463
RF-500	0.7200	0.7903	0.7200	0.7535
RF-1000	0.7210	0.7893	0.7210	0.7536

The test set is composed of 1000 images (500 COVID, 500 non-COVID)

From Table 10, we note that the shallow algorithms have an inferior performance. On the other hand, the HB-DDCAE method has the same performance of the proposed approach.

### Test-time comparisons

Table 11 reports the (numerically evaluated) average times required by the implemented methods for classifying a batch of 10 images in the test phase. A comparison of the entries of this table leads to the conclusion that the average test time of our method is over $25\%$ less than the corresponding ones of the implemented benchmark models. We have numerically ascertained that this is due to the fact that our proposed method works on the reduced-size (i.e. compressed) hidden feature vectors, while all the benchmark models directly process the full-size input test images. This conclusion provides further support about the actual effectiveness of the proposed KDE-based classifying approach.

**Table 11:** Average test times over a batch of 10 images

Model	Test time $\left( s\right)$
AlexNet	1.3566
GoogleNet	1.2400
ResNet18	2.4754
Proposed	1.0026

## Conclusion and hints for future research

We propose a method for classifying lung CTs as COVID-19 or non-COVID-19. The method exploits a DCAE trained on COVID-19 CTs only and a KDE estimation of the PDF of the DCAE hidden feature vectors. The DCAE is used to produce the corresponding hidden feature vector to classify an image. Afterwards, we use the so-obtained PDF evaluation to compute, in the test phase, the PDF value of the hidden feature vector, corresponding to a test image: if the PDF value is above a suitable threshold, that image is classified as COVID-19, otherwise as non-COVID-19.

We compare our KDE-based approach to the benchmark method that is based on the reconstruction error. In addition, we also check the accuracy performance of three widely known supervised models and the results of some recent papers. The carried out tests support the conclusion that the proposed approach is highly effective in terms of both achieved test accuracy and the needed test times.

The presented results could be extended, at least, along two main research lines. A first research line could concern the utilisation of generative adversarial networks (GANs) for the generation of additional training examples in the case of new COVID-19 mutations (as, for example, the Omicron one), in order to quickly provide reliable automatic detection of these mutations without pausing for the acquisition of sufficiently large new datasets. A second research line could regard the implementation of the proposed algorithmic framework atop distributed Fog/Cloud networked technological platforms [50, 51], in order to be capable to quickly generate reliable clinical diagnosis by leveraging the low-delay and (possibly, adaptive [52] and /or multiantenna empowered [53, 54]) capability of emerging Fog computing platforms for allowing ubiquitous wireless access to computing-demanding medical diagnostic services.

## Footnotes

1 The KDE is a traditional density estimation method. Such methods may become unmanageable when the number of variables is too high. Luckily, the computational power of modern computers makes this method suitable for the problem at hand.
